# Association between Phenotypic Age and Mortality in Patients with Multivessel Coronary Artery Disease

**DOI:** 10.1155/2022/4524032

**Published:** 2022-01-13

**Authors:** Qiong Ma, Bo-Lin Li, Lei Yang, Miao Zhang, Xin-Xin Feng, Qian Li, Hui Liu, Ya-Jie Gao, Wen-Zhuo Ma, Rui-Juan Shi, Yan-Bo Xue, Xiao-Pu Zheng, Ke Gao, Jian-Jun Mu

**Affiliations:** ^1^Department of Cardiology, The First Affiliated Hospital of Xi'an Jiaotong University, Xi'an, Shaanxi, China; ^2^Key Laboratory of Molecular Cardiology of Shaanxi Province, Xi'an, Shaanxi, China; ^3^Department of Ultrasound, The Second Affiliated Hospital of Xi'an Jiaotong University, Xi'an, Shaanxi, China; ^4^Department of Oncology Radiology, The Second Affiliated Hospital of Xi'an Jiaotong University, Xi'an, Shaanxi, China; ^5^Biobank, The First Affiliated Hospital of Xi'an Jiaotong University, Xi'an, Shaanxi, China; ^6^Department of Biomedical and Pharmaceutical Sciences, University of Rhode Island, College of Pharmacy, Rhode Island, USA

## Abstract

**Background:**

Chronological age (CA) is not a perfect proxy for the true biological aging status of the body. A new biological aging measure, phenotypic age (PhenoAge), has been shown to capture morbidity and mortality risk in the general US population and diverse subpopulations. This study was aimed at evaluating the association between PhenoAge and long-term outcome of patients with multivessel coronary artery disease (CAD).

**Methods:**

A total of 609 multivessel CAD patients who received PCI attempt and with follow-up were enrolled. The clinical outcome was all-cause mortality on follow-up. PhenoAge was calculated using an equation constructed from CA and 9 clinical biomarkers. Cox proportional hazards regression models and receiver operating characteristic (ROC) curves were performed to evaluate the association between PhenoAge and mortality.

**Results:**

Overall, patients with more diseases had older PhenoAge and phenotypic age acceleration (PhenoAgeAccel). After a median follow-up of 33.5 months, those with positive PhenoAgeAccel had a significantly higher incidence of all-cause mortality (*P* = 0.001). After adjusting for CA, Cox proportional hazards models showed that both PhenoAge and PhenoAgeAccel were significantly associated with all-cause mortality. Even after further adjusting for confounding factors, each 10-year increase in PhenoAge was also associated with a 51% increased mortality risk. ROC curves revealed that PhenoAge, with an area under the curve of 0.705, significantly outperformed CA, the individual clinical chemistry measure, and other risk factors. When reexamining the ROC curves using various combinations of variables, we found that PhenoAge provides additional predictive power to all models.

**Conclusions:**

In conclusion, PhenoAge was strongly associated with all-cause mortality even after adjusting for CA. Our findings suggest that PhenoAge measure may be complementary in predicting mortality risk for patients with multivessel CAD.

## 1. Introduction

Aging is the major contributing factor to most chronic noncommunicable diseases, such as hypertension, coronary artery disease (CAD), and chronic respiratory diseases [1–3]. However, persons with the same chronological age (CA) may have considerably diverse susceptibilities to diseases and death, which may reflect differences in their biological aging processes. Therefore, CA does not sufficiently reflect the underlying state of biological aging and functional degradation [4]. Aging encompasses systemic, multiple organ-level, cellular, and molecular changes that predispose individuals to cardiovascular and comorbid conditions [5]. Scientists are now looking at the physiological changes along the aging course to better predict who is at the greatest risk of illnesses and death [6, 7]. Phenotypic age (PhenoAge), a novel biological aging measure, has been shown to capture morbidity and mortality risk across diverse subpopulations that include healthy and unhealthy individuals, distinct age groups, and patients with heart disease [8–11]. Phenotypic age acceleration (PhenoAgeAccel), an aging biomarker, represents PhenoAge after accounting for CA. More importantly, PhenoAge can differentiate mortality risk among persons at the same CA, it may serve as a useful tool for evaluating intervention efficacy in clinical research, avoiding the need for decades of follow-up [8]. However, the applicability of this new aging measure across diverse subpopulations with specific disease or other cohorts remains to be further evaluated.

Advancing age is the strongest risk factor related to the development of cardiovascular disease, and CAD is a major cause of morbidity and mortality in older patients [12, 13]. Necropsy studies have reported a high prevalence of obstructive CAD in older patients, often with characteristics of multivessel disease, severe calcification, and tortuosity [12]. However, in clinical practice, an individual with same CA or the same risk factor profiles may exhibit differences in susceptibility to cardiovascular diseases and death [14]. The reasons for this wide interindividual variation in susceptibility are poorly understood. Several studies have shown that biological aging may be involved in the onset of cardiovascular disease and mortality [4, 9, 15]. Hence, understanding the biologic processes of aging and how these processes confer susceptibility to CAD and mortality may lead to successful treatments and interventions that lessen aging and reduce the burden of cardiac diseases [8, 15].

To our knowledge, the relationship between PhenoAge and mortality in terms of specific cardiovascular disease and the differences between individuals based on their PhenoAge acceleration (PhenoAgeAccel) remain unclear. To date, there is no evidence that PhenoAge is highly predictive of mortality risk among patients with multivessel CAD than CA. Therefore, this study attempts to answer these important questions.

## 2. Methods

### 2.1. Study Population

Patients with coronary artery lesions were confirmed by coronary angiography in the Cardiology Department of the First Affiliated Hospital of Xi'an Jiaotong University (Xi'an, Shaanxi, China) between June 2013 and October 2017. The inclusion criteria for the present study were (1) symptomatic angina and/or functional ischemia, including unstable angina pectoris and stable angina pectoris; (2) two- or three-vessel coronary artery lesions detected on diagnostic coronary angiography. Exclusion criteria were (1) history of cardiogenic shock or cardiopulmonary resuscitation; (2) physician-diagnosed chronic diseases in medical records, including severe arrhythmia (atrial fibrillation, ventricular tachycardia, and permanent cardiac pacemaker), malignant tumor, depression, Alzheimer's disease, Parkinson's disease, acute myocardial infarction, acute stroke, or infectious diseases; (3) previous coronary artery bypass graft surgery; (4) patients with single coronary artery lesions; and (5) patients who were lost to follow-up. The follow-up for all-cause and cardiovascular mortality was carried out via medical records, outpatient visit contacts, or telephone between 2018 and 2019. Finally, a total of 609 multivessel CAD patients who underwent PCI attempt and complete follow-up were included in the study. The detailed selection process is shown in Figure S1. The study was approved by both Research and Ethics Committees of the First Affiliated Hospital of Xi'an Jiaotong University, and all participants were required to provide written informed consent.

### 2.2. Definitions

Eligible patients had multivessel CAD defined as at least two coronary artery lesions that was (1) in a vessel with diameter of at least 2.5 mm; (2) at least 70% diameter stenosis by visual estimation or, alternatively, 50–69% diameter stenosis by visual estimation and a FFR measurement of less than or equal to 0.80; and (3) planned revascularization with PCI [16]. Two different revascularization strategies are available for the treatment of multivessel disease at the time of PCI: (1) complete revascularization strategy (all lesions with complete revascularization at the index procedure or as a staged procedure either during the hospitalization or within a few weeks after discharge); (2) incomplete revascularization strategy (only culprit artery was treated at the index procedure) [17]. Technical success was defined as residual stenosis of less than 30% with antegrade flow TIMI 3 and accompanied by complete revascularization strategy. Procedural success was defined as technical success plus no in-hospital adverse cardiac effects, including myocardial infarction (MI), all-cause mortality, and recurrence of cardiac symptoms requiring repeat vessel revascularization [18]. The primary outcomes were all-cause mortality and cardiac death on follow-up [19, 20]. According to the medical records, during hospitalization, chronic diseases included five common coexisting cardiometabolic, kidney, and cerebrovascular diseases: CAD, hypertension, type 2 diabetes, stroke, and chronic kidney disease. Based on the disease count, we created a variable with three categories: one disease (CAD alone), two diseases, and three or more diseases.

### 2.3. Demographic Characteristics and Clinical Parameters

We collected several demographic, clinical, and analytical parameters based on the medical records. Fasting peripheral blood samples were collected from all patients within 24 hours of admission. Several biomarkers, including total white blood cells, leukocyte subtype counts, serum uric acid, albumin, creatinine, blood glucose, lipid levels, C-reactive protein, alkaline phosphatase, creatine kinase, creatine phosphokinase isoenzyme, and N-terminal probrain natriuretic peptide were measured. The estimated glomerular filtration rate was calculated according to the Chronic Kidney Disease Epidemiology Collaboration's 2009 creatinine equation [21].

### 2.4. Phenotypic Age and Phenotypic Age Acceleration

According to the method established in 2018 [8], PhenoAge was calculated retrospectively based on data collected on clinical records. In brief, PhenoAge was developed using CA and nine clinical biomarkers, including albumin, creatinine, glucose, ln (C-reactive protein), lymphocyte percent, mean cell volume, red blood cell distribution width, alkaline phosphatase, and white blood cell count [22]. The equation for calculating PhenoAge was based on two parametric proportional hazards models, and the score was transformed into units of years. For instance, an individual may be 50 years old chronologically, but he/she has a PhenoAge of 55 years, indicating that he/she has the average morbidity and/or mortality risk of someone who is 55 years old chronologically. In addition, PhenoAgeAccel was calculated according to the residual resulting from a linear model when regressing PhenoAge on CA. Therefore, it represents PhenoAge after accounting for CA. PhenoAgeAccel reflects whether a person's biological age is older (positive value) or younger (negative value) than expected based on his/her CA.

### 2.5. Statistical Analysis

Data are presented as means ± standard deviation or median and interquartile range for continuous variables and percentages for categorical variables. Continuous variables that had a normal distribution were evaluated using analysis of variance, whereas the Kruskal-Wallis test was used for nonnormally distributed data. Pearson correlation analysis was used to analyze the relationship between PhenoAge and CA. Based on the disease count categories, we then compared the PhenoAge and PhenoAgeAccel value among the full sample.

Next, patients were then divided into two subgroups for PhenoAgeAccel, so that the positive PhenoAgeAccel subgroup represented individuals who were most at risk of adverse events for their age. We then analyzed the long-term outcomes for subjects in the positive versus negative subgroup. The clinical outcome occurring over time for all-cause mortality was described by Kaplan-Meier survival curves and compared by the log-rank test. Moreover, Cox proportional hazards regression models were performed to evaluate the relationships between PhenoAge and mortality. In the end, ROC curves were used to compare the all-cause mortality risk prediction of PhenoAge to predictions based on traditional risk assessment tools (e.g., based on CA, lesion number, revascularization strategy, disease counts, and biomarkers). DeLong's test was used to compare the AUC of each variable (MedCalc version 19.1, Belgium). All statistical analysis was performed retrospectively with SPSS 25.0 (SPSS, Inc., Chicago, IL, USA) and R version 3.6.3. In all cases, *P* < 0.05 was considered significant.

## 3. Results

### 3.1. Study Population and Clinical Characteristics

The characteristics of the study patients are shown in Table 1. The mean CA of the 609 patients was 65.9 years, and 83.1% of patients were males. Approximately 28.4% of the study participants had CAD alone at admission, while 39.4% reported having been diagnosed with two chronic diseases, and 32.2% reported at least three coexisting chronic diseases. After a median follow-up of 33.5 (interquartile range: 23 to 48) months, the overall number of all-cause mortality and cardiovascular mortality was 62 (10.2%) and 32 (5.3%), respectively.

### 3.2. PhenoAge and PhenoAgeAccel according to Disease Count

**Figure 1:**
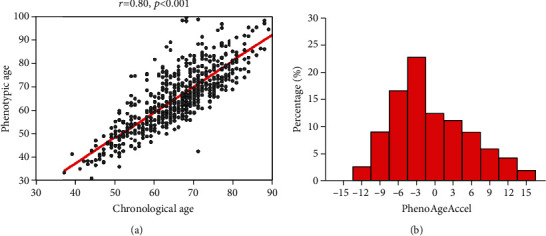
Relationship between PhenoAge, CA, and PhenoAgeAccel. (a) As expected, PhenoAge was highly correlated with CA (*r* = 0.80, *P* < 0.001). The red line depicts the expected PhenoAge for each CA, with points above the line depicting CAD patients who were phenotypically older than expected and points below the line depicting those who were phenotypically younger than expected. (b) PhenoAgeAccel was not fairly normally distributed, with a median of -1.69 (interquartile range: -5.02 to 4.51), and 41.7% (254/609) of patients tend to be in the positive (older) direction. CA: chronological age; PhenoAge: Phenotypic Age. PhenoAgeAccel: phenotypic age acceleration.

**Figure 2:**
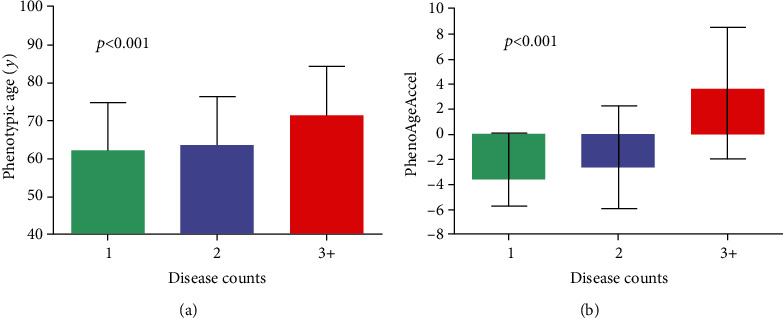
PhenoAge and PhenoAgeAccel for each disease count. The *y-*axis depicts the mean and standard deviation of (a) PhenoAge and the median and interquartile range of (b) PhenoAgeAccel; the *x-*axis shows groups categorized based on the number of chronic diseases each participant had.

Figure 1 shows the correlation between PhenoAge and CA, as well as the distribution of PhenoAgeAccel. PhenoAge is highly associated with CA (*r* = 0.80, *P* < 0.001). We calculated the residual for PhenoAge, referred to as PhenoAgeAccel. While PhenoAgeAccel was not normally distributed, with a median of -1.69 (interquartile range: -5.02 to 4.51), 41.7% (254/609) of patients tend to be in the positive (older) direction.  Figure 2 shows PhenoAge and predicted increases in PhenoAgeAccel for each disease count category. Overall, patients with three or more chronic diseases had older PhenoAge compared to those with two or less diseases (*P* for trend <0.001, Figure 2(a)). Moreover, we observed that PhenoAgeAccel was highest among patients who were diagnosed with three or more chronic diseases (*P* for trend<0.001, Figure 2(b)). The median (interquartile range) of PhenoAgeAccel was -3.55 (-5.69, 0.05) years among participants with one disease, -2.66 (-5.91, 2.26) years among patients with two diseases, and 3.57 (-2.00, 8.48) years among participants with three or more diseases. Among these CAD patients, those with two chronic diseases were on average 1.61 years older phenotypically than persons with CAD alone (-1.00 vs. -2.61 years, *P* = 0.031), and those with three or more diseases were about 6.66 years older phenotypically (4.05 vs. -2.61 years, *P* < 0.001).

### 3.3. Associations between PhenoAge, PhenoAgeAccel, and Mortality

**Figure 3:**
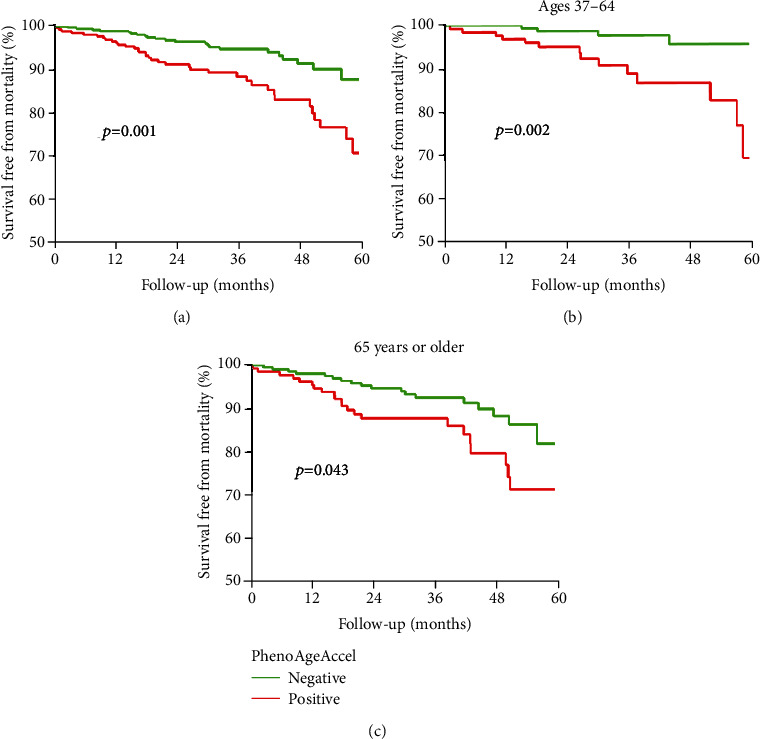
Kaplan–Meier curves for all-cause mortality in the positive versus the negative PhenoAgeAccel subgroup. Kaplan-Meier curves describing the risk of all-cause mortality according to baseline PhenoAgeAccel.

Table 2 shows the relationships between PhenoAge, PhenoAgeAccel, and clinical outcomes, based on multivariable COX proportional hazards models. PhenoAgeAccel was calculated by regression residual between PhenoAge and CA. As a result, when corrected for CA, both models are functionally identical if PhenoAge (per year) and PhenoAgeAccel (per year) are included as predictors. After adjusting for CA, both PhenoAge and PhenoAgeAccel (HR:1.05, 95% CI: 1.02–1.08) were significantly associated with all-cause mortality. Even after further adjusting for several demographics, clinical and analytical parameters, and other risk factors, PhenoAge was positively associated with all-cause mortality on follow-up. And each 10-year increase in PhenoAge was associated with a 51% increased mortality risk (HR:1.51, 95% CI: 1.10–2.08; *P* < 0.05). Next, all patients were then divided into two subgroups according to PhenoAgeAccel, so that the positive PhenoAgeAccel subgroup individuals (*n* = 254) were most at risk of adverse events for their age. As shown in Figure 3, we found that those with positive PhenoAgeAccel relative to their chronological ages had a significantly higher incidence of all-cause mortality in both the full sample and sample based on age stratification (all *P* < 0.05). After adjusting for confounding factors, individuals with positive PhenoAgeAccel were more likely to have incident mortality risk than negative PhenoAgeAccel peers (HR: 2.08; 95% CI: 1.14–3.80, Table 2).

**Figure 4:**
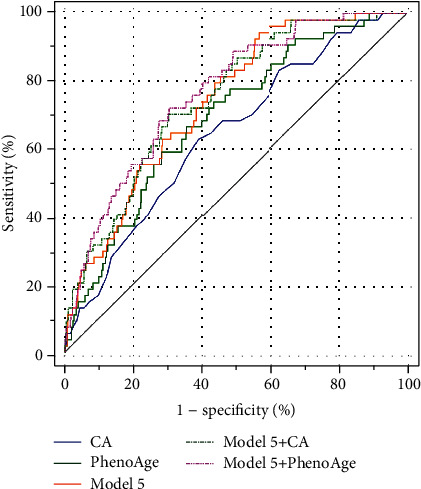
Receiver-operating characteristic curves for all-cause mortality. Receiver-operating characteristic curve presents that PhenoAge predicted risk of mortality better than CA (AUC: 0.705 vs. 0.654, *P* = 0.040). It was only when PhenoAge, demographics, clinical and analytical parameters, and disease count were all included in a single model (model 5+PhenoAge) that the AUC started to significantly exceed the AUC for PhenoAge alone (AUC: 0.765 vs. 0.705, *P* = 0.002). AUC: area under the curve; CA: chronological age; PhenoAge: phenotypic age; model 5: a model that includes lesion number, revascularization and disease counts, gender, smoking, drinking, body mass index, serum uric acid, creatine kinase, creatine phosphokinase isoenzyme, total cholesterol, triglycerides, low-density lipoprotein cholesterol, high-density lipoprotein cholesterol, and N-terminal probrain natriuretic peptide.

Receiver operating characteristic (ROC) curve analysis was performed to compare the predictive potential of PhenoAge with CA, lesion number, revascularization, disease count, several clinical and analytical parameters, and various combinations of variables. ROC curves for all-cause mortality revealed that PhenoAge, with an area under the curve (AUC) of 0.705, outperformed CA (Figure 4 and Table 3). And PhenoAge alone was more predictive of 3-year all-cause mortality than the individual clinical biomarker and other traditional cardiovascular risk factors, including lesion number, revascularization, and disease count. As shown in Table 3 and Figure 4, when reexamining the ROC curves using various combinations of variables (in model 4 and model 5), with and without PhenoAge included, we found that PhenoAge added more predictive power to all models than CA.

We also assessed the association between PhenoAge and cardiovascular mortality. After a median follow-up of 33.5 months, the number of cardiovascular mortality cases was 32 (5.3%). As shown in Supplementary Table 1, we found that each one-year increase in PhenoAge was significantly associated with a 5% increased cardiovascular mortality risk (HR:1.05, 95% CI: 1.01–1.10). More interestingly, PhenoAge alone tends to be more predictive of cardiovascular mortality than CA and model 4 that included lesion number, revascularization, and disease count (Supplementary Table 2).

## 4. Discussion

To the best of our knowledge, the present study is the first one to evaluate the role of this new biological aging measure, PhenoAge, in predicting mortality of patients with multivessel CAD. The findings of the study were as follows: (1) PhenoAge was highly predictive of all-cause mortality even after adjusting for CA; (2) as expected of an aging biomarker, PhenoAgeAccel also tracks multimorbidity and risk of mortality on follow-up; (3) PhenoAge captures something above and beyond what can be explained for mortality risk by CA, individual clinical chemistry measures, and other traditional risk factors.

A person's rate of aging may be able to influence his/her susceptibility to morbidity and mortality [10]. Therefore, identification of individuals at higher risk of cardiovascular disease and mortality is key to extend health span in the light of the escalating burden of aging populations worldwide. At present, plenty of traditional cardiovascular risk factors including CA are available to assess the risk of coronary heart disease and cardiac mortality [12, 23–25]. However, CA refers only to the passage of time; it does not reflect the underlying state of physiological breakdown along the aging process [9]. Biological aging relates to decline in function, and it is a complex gradual process which is highly variable in health at a given CA [4, 7].

Biological age can be a novel and useful marker to recognise high-risk populations who are most susceptible to specific disease and death with increasing CA. It has been reported that several methods can be used to measure biological age, such as DNA methylation age and the recombination of several biomarkers [9, 26–29]. However, the fields of biological aging and cardiovascular medicine are still largely separated. Recently, some studies reported that cardiovascular disease and aging were highly interconnected and may share common pathways [30, 31]. Many of the factors underlying aging-related changes in the vascular system and myocardium are also implicated in the development of cardiac diseases, such as endothelial dysfunction, coronary arterial stiffness, perivascular and myocardial fibrosis, and loss of functional cardiac cells [26, 30, 32]. Marioni et al. reported that DNA methylation-derived measures of accelerated biological aging were heritable traits that predict mortality independently of CA and other known genetic factors [28]. Telomere length is often used as a cellular marker for biological age. A growing body of evidence has demonstrated that telomere length is inversely associated with cardiovascular disease, including CAD, hypertension, heart failure, and death [14, 33, 34]. Recently, Liu et al. showed that PhenoAge measurements can stratify multimorbidity and mortality risks among US population [8]. The advantage of PhenoAge equation is that it is based on continuous measurement of physiological indicators with standard techniques, so it is not easily affected by changes in measurement methods.

In this study, we investigated the predictive value of PhenoAge for clinical outcomes in CAD patients after a median follow-up of nearly 3 years. We found that PhenoAge is still a useful predictor of mortality after accounting for CA. This suggests that PhenoAge captures the effects of biological aging even when cardiac disease become clinically evident. Despite relatively small sample sizes, we still observed that PhenoAgeAccel can be used to stratify multimorbidity and mortality risk among CAD patients. Both PhenoAge and PhenoAgeAccel were increased with the number of chronic disorders, suggesting that the more coexisting chronic conditions a CAD patient has, the phenotypically older he appears. Moreover, ROC curve presents that PhenoAge predicted risk of all-cause death better than CA, lesion number, disease counts, and revascularization strategy as well as other risk factors. When reexamining the ROC curves using various combinations of variables, we found that PhenoAge provides additional predictive power to all models. These finding suggest that PhenoAge captures something above and beyond what can be explained for mortality risk by demographics, clinical and analytical parameters, disease, and other individual's conventional cardiovascular risk factors. However, PhenoAge does not add very much above and beyond CA for mortality risk predictions, especially in model 5 (AUC for model 5+PhenoAge vs. model 5+CA: 0.765 vs. 0.748). The reasons for this result may be as follows: (1) some traditional CAD risk factors, such as blood lipids, serum uric acid, and body mass index, have been proven to be powerful risk factors for death; thus, the addition of these risk factors may offset or bias the predictive power of PhenoAge for mortality; (2) each 1-year increase in PhenoAge or CA is unlikely to result in a substantially increased risk of death, as this may not be consistent with what is observed in clinical practice. Many chronic diseases and declined physical function that occur/develop with age, rather than age alone, are the main causes of death [1]. Therefore, the addition of phenotypic age alone is unlikely to significantly increase its predictive power for all-cause mortality in all models (model 4 and model 5).

Although PhenoAge cannot replace the well-established risk prediction system for cardiovascular diseases, it may serve as a useful tool to facilitate identification of at-risk individuals and evaluation of the efficacy of cardiovascular treatments. As we know, early identification of high-risk patients is the focus of clinical research related to biomarkers. Emerging evidence shows that the incidence of cardiovascular diseases has been either steady or increasing among young adults over the past 2 decades in contrast to the downward trend in older adults [35]. One of the reasons is that patients with lower CA are often considered as low-risk groups, thus missing the opportunities for early diagnosis and treatment. In conventional cardiovascular risk stratification, the screening and identification of young high-risk patients may be restricted by their CA. In fact, CA is often nonlinearly correlated with risk of mortality. The younger the patient is, the lower the predictive value of CA for mortality. At the same time, the predictive value of risk factors that are collinear with CA is also weakened. In our study, differences in survival rate free from all-cause mortality between the negative PhenoAgeAccel and positive PhenoAgeAccel groups in the middle-aged group were larger than those in the older CA group. Liu et al. also found that in young adults, PhenoAge is still independently correlated with all-cause mortality [8]. These results indicate that PhenoAge has potential application in all age groups. In addition, physicians may be able to delay the progression of PhenoAge by strengthening clinical interventions, thereby reducing the risk of death, so that PhenoAge has the potential to play a role in the long-term follow-up as an indicator of efficacy evaluation. Further clinical trials are needed to support this hypothesis.

The pathophysiological mechanism of such relationship between PhenoAge and mortality of patients with multivessel CAD may be as follows: first, advancing age is positively associated with decreased regeneration ability of endothelial cell and endothelial senescence [26, 36, 37]. Histologically, cell senescence is the main hallmark of human atherosclerosis, and it promotes the synthesis of several intracellular and secreted proteins related to plaque development and stability [26, 30, 36]; second, chronic inflammation is an indispensable factor for both aging and the initiation and progression of atherosclerosis. Compelling evidence showed that elevated inflammatory markers, such as tumor necrosis factor, C-reactive protein, and interleukin-6, represent the activation of coronary artery damage and inflammaging [26, 38]. And inflammaging is believed to carry high susceptibility to frailty, biological aging, cardiovascular disease, multimorbidity, and premature death [6, 26, 39]. Therefore, at least to some extent, interindividual variation in risk of CAD and mortality might result from variation in the rate of biological aging.

The present study has several strengths. Firstly, this is the first study to describe the application of a new biomarker for biological aging to the clinical problem of CAD and the prediction of mortality of that disease. Secondly, the results could be relevant for clinical practice, especially because we try to combine some gerontological concepts (PhenoAge, PhenoAgeAccel) with cardiology practice. Given the high and rising prevalence of CAD and the urgent need to adjust allocation of limited resources of follow-up-monitoring to individual risk, it is to be expected that this approach could indeed have a significant impact on disease management in CAD.

Our study has several limitations. First of all, it shares all the limitations of observational, single-center studies, and there was lack of longitudinal data for PhenoAge, so we did not calculate the pace of aging that could further confirm the rate of change in PhenoAge (true acceleration). Secondly, this was a retrospective study unpowered to identify all changes to patients' medical treatment strategies and routine angiographic during follow-up; thus, we were unable to evaluate the real impact of the rate of change in PhenoAge on in-stent restenosis or long-term outcomes of patients with multivessel CAD. Besides, this study used observational data with small sample sizes, which may have biased the observed relations by introducing confounding factors. To reduce such bias, we considered as many related factors as possible in the analysis; however, other potential confounding factors, such as disability, cognitive function, physical inactivity, and SYNTAX score, cannot be ruled out. More importantly, we only focused on patients with multivessel CAD in the study, so the limited clinical applicability should also be considered when interpreting and extrapolating our results. Future studies are needed to pinpoint the role of PhenoAge in other cohorts or different subpopulations with specific disease. Because of these limitations, further work is needed to further develop the biological age construct and its application to identify and monitor individuals at higher risk of cardiovascular disease and mortality at a later age, offering opportunities for primary prevention and evaluation of intervention efficacy.

## 5. Conclusions

In conclusion, PhenoAge was associated with all-cause mortality even after adjusting for CA. Our findings suggest that PhenoAge measure may be complementary in predicting mortality risk for patients with multivessel CAD.

## Figures and Tables

**Table 1:** Characteristics of the study participants.

Characteristics	No. (%) or mean ± SD
All	609
Chronological age (y)	65.9 ± 9.8
Men, *n* (%)	506 (83.1)
Body mass index, kg/m^2^	24.3 ± 3.1
Current smoker, *n* (%)	298 (48.9)
Drinking, *n* (%)	132 (21.7)
Physician-diagnosed diseases	
Hypertension	342 (56.2)
Type 2 diabetes	224 (36.8)
Stroke	61 (10.0)
Chronic kidney disease	46 (7.6)
CAD	609 (100)
Disease count, *n* (%)	
1	173 (28.4)
2	240 (39.4)
3+	196 (32.2)
Oral medications, *n* (%)	
Antiplatelet drugs	475 (78.0)
Statins	371 (60.9)
Beta-blockers	319 (52.4)
ACEI/ARB	165 (27.1)
Calcium channel blocker	130 (21.3)
Nitrate	65 (10.7)
Preoperative laboratory measurements	
Hemoglobin (g/L)	136.2 ± 18.4
Red cell distribution width (%)	13.4 ± 1.0
White blood cell (10^9^/L)	6.7 ± 2.1
Lymphocyte percent (%)	24.8 ± 8.0
Mean cell volume (fL)	94.1 ± 0.6
Albumin (mg/dL)	37.6 ± 3.6
Glucose (mmol/L)	6.6 ± 2.6
Serum uric acid (*μ*mol/L)	346.4 ± 92.4
Creatinine (*μ*mol/L)	74.0 ± 20.5
Alkaline phosphatase (U/L)	81.8 ± 25.1
Total cholesterol (mmol/L)	3.8 ± 0.9
Total triglycerides (mmol/L)	1.8 ± 1.2
Low-density lipoprotein cholesterol (mmol/L)	2.2 ± 0.8
High-density lipoprotein cholesterol (mmol/L)	1.0 ± 0.4
C-reactive protein (mg/dL)	0.4 (0.3, 0.8)
Creatine kinase (U/L)	73 (57, 105)
Creatine phosphokinase isoenzyme (U/L)	12 (9, 16)
N-terminal probrain natriuretic peptide (pg/mL)	355 (150, 1085)
PhenoAge (y)	65.6 ± 13.4
PhenoAgeAccel (y)	-1.7 (-5.0, 4.5)
PhenoAgeAccel subgroups	
Negative, *n* (%)	355 (58.3)
Positive, *n* (%)	254 (41.7)
Angiographic and procedural characteristics	
Number of diseased vessels, *n* (%)	
2	117 (19.2)
3	492 (80.8)
Procedural success, *n* (%)	336 (55.2)
Clinical outcomes on follow-up	
All-cause mortality, *n* (%)	62 (10.2)
Cardiac mortality, *n* (%)	32 (5.3)
Others, *n* (%)	30 (4.9)

ACEI: angiotensin-converting enzyme inhibitors; ARB: angiotensin receptor blockers; PhenoAge: Phenotypic Age; PhenoAgeAccel: phenotypic age acceleration.

**Table 2:** Associations between PhenoAge, PhenoAgeAccel, and all-cause mortality on follow-up.

Variable	HR (95% CI)		
	Model 1^a^	Model 2^b^	Model 3^c^
PhenoAge (per year)	1.05 (1.02, 1.08)^∗∗∗^	1.06 (1.03, 1.09)^∗∗∗^	1.04 (1.01, 1.08)^∗^
PhenoAge (per 10 years)	1.63 (1.27, 2.11)^∗∗∗^	1.74 (1.34, 2.27)^∗∗∗^	1.51 (1.10, 2.08)^∗^
PhenoAgeAccel subgroups			
Negative	Reference	Reference	Reference
Positive	2.29 (1.38, 3.81)^∗∗^	2.58 (1.51, 4.41)^∗∗^	2.08 (1.14, 3.80)^∗^

Results are based on COX regression analysis. HR: hazard ratio; PhenoAge: phenotypic age; PhenoAgeAccel: phenotypic age acceleration. ^a^Model 1 adjusted for chronological age. ^b^Model 2 additionally adjusted for lesion number, disease count, and revascularization. ^c^Model 3 additionally adjusted for other traditional cardiovascular risk factors, including gender, smoking, drinking, body mass index, serum uric acid, creatine kinase, creatine phosphokinase isoenzyme, total cholesterol, triglycerides, low-density lipoprotein cholesterol, high-density lipoprotein cholesterol, and N-terminal probrain natriuretic peptide. ^+^*P* < 0.1; ^∗^*P* < 0.05; ^∗∗^*P* < 0.01; ^∗∗∗^*P* < 0.001.

**Table 3:** Area under the curve for all-cause mortality.

Variable	AUC	SE	*P* value	*Z* value	*P* for comparison
PhenoAge	0.705	0.033	0.000	Reference	Reference
CA	0.654	0.036	0.000	2.058	0.040
Lesion number	0.517	0.025	0.658	4.479	0.000
Revascularization	0.529	0.039	0.457	3.856	0.000
Disease counts	0.523	0.037	0.304	4.123	0.000
Serum uric acid	0.508	0.043	0.839	3.352	0.000
Total cholesterol	0.487	0.039	0.739	4.174	0.000
Total triglycerides	0.448	0.037	0.176	3.368	0.001
LDL-c	0.474	0.040	0.498	3.808	0.000
HDL-c	0.498	0.041	0.954	3.766	0.000
Creatine kinase	0.428	0.039	0.071	2.769	0.006
CK-MB	0.529	0.038	0.470	3.237	0.001
Model 4	0.540	0.041	0.298	3.382	0.001
Model 4*+* CA	0.662	0.035	0.000	1.703	0.089
Model 4+ PhenoAge	0.709	0.033	0.000	0.465	0.642
Model 5	0.740	0.031	0.000	1.233	0.218
Model 5+ CA	0.748	0.032	0.000	1.861	0.063
Model 5+ PhenoAge	0.765	0.031	0.000	3.141	0.002

AUC: area under the curve; SE: standard error; PhenoAge: phenotypic age; CA: chronological age; LDL-c: low-density lipoprotein cholesterol; HDL-c: high-density lipoprotein cholesterol; CK-MB: creatine phosphokinase isoenzyme. Model 4: a model that includes lesion number, revascularization, and disease count. Model 5: a model that includes all the variables in model 4, as well as gender, smoking, drinking, body mass index, serum uric acid, creatine kinase, creatine phosphokinase isoenzyme, total cholesterol, triglycerides, low-density lipoprotein cholesterol, high-density lipoprotein cholesterol, and N-terminal pro-brain natriuretic peptide.

## Data Availability

The datasets generated for this study are available on request to the corresponding author.

## Supplementary Materials

Supplementary Materials Figure S1: flow diagram for participants included in the study. Supplementary Table 1: association between PhenoAge and cardiovascular mortality. Supplementary Table 2: area under the curve for cardiovascular mortality.
