# Supplementary material for: Association between Phenotypic Age and Mortality in Patients with Multivessel Coronary Artery Disease
Source: Dis Markers. 2022 Jan 13;2022:4524032. doi: 10.1155/2022/4524032 (PMC8776473; doi:10.1155/2022/4524032)
Supplement: Supplementary Materials — Figure S1: flow diagram for participants included in the study. Supplementary Table 1: association between PhenoAge and cardiovascular mortality. Supplementary Table 2: area under the curve for cardiovascular mortality. [file 4524032.f1.docx]

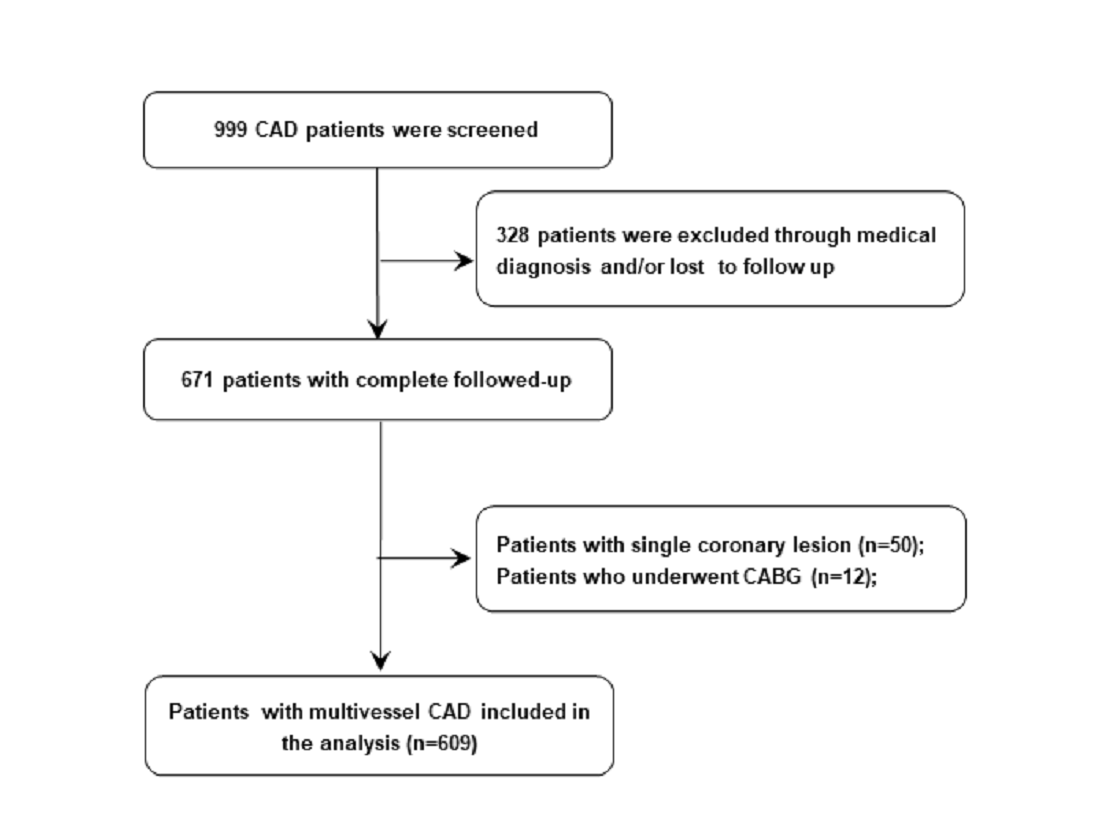


**Figure S1. Flow diagram for participants included in the study**

Abbreviation: CAD: coronary artery disease; CABG: coronary artery bypass graft surgery.

**Supplementary Table 1 Association between PhenoAge and cardiovascular mortality**

| **Variable** | **HR (95% CI)** | | |
| --- | --- | --- | --- |
|  | **Model 1^a^** | **Model 2^b^** | **Model 3^c^** |
| PhenoAge (per year) | 1.05 (1.02, 1.09) ^**^ | 1.06 (1.02, 1.11) ^**^ | 1.05 (1.01, 1.10) ^*^ |
| PhenoAge (per 10 years) | 1.66 (1.17, 2.37) ^**^ | 1.81 (1.23, 2.66) ^**^ | 1.62 (1.02, 2.57) ^*^ |
| PhenoAgeAccel subgroups |  |  |  |
| Negative | Reference | Reference | Reference |
| Positive | 2.01 (1.00, 4.04) ^+^ | 2.17 (1.03, 4.57) ^*^ | 1.84 (0.79, 4.32) |

Results are based on COX regression analysis. HR: hazard ratio; PhenoAge: Phenotypic Age; PhenoAgeAccel: Phenotypic Age Acceleration.

**^a^** Model 1 adjusted for chronological age;

**^b^** Model 2 additionally adjusted for lesion number, disease count and revascularization;

**^c^** Model 3 additionally adjusted for other traditional cardiovascular risk factors, including gender, smoking, drinking, body mass index, serum uric acid, [creatine](javascript:;) [kinase](javascript:;), creatine phosphokinase isoenzyme, total cholesterol, triglycerides, low-density lipoprotein cholesterol, high-density lipoprotein cholesterol and N-terminal pro-brain natriuretic peptide.

^+^*P <* 0.1; ^*^*P <* 0.05; ^**^*P <* 0.01; ^***^*P <* 0.001.

**Supplementary Table 2 Area under the curve for cardiovascular mortality**

| **Variable** | **AUC** | **SE** | ***P* value** | **Z value** | ***P* for comparison** |
| --- | --- | --- | --- | --- | --- |
| PhenoAge | 0.679 | 0.050 | 0.001 | Reference | Reference |
| CA | 0.639 | 0.055 | 0.008 | 1.458 | 0.144 |
| Model 4 | 0.635 | 0.049 | 0.010 | 0.636 | 0.525 |
| Model 4*+* CA | 0.675 | 0.051 | 0.001 | 0.076 | 0.939 |
| Model 4+ PhenoAge | 0.707 | 0.045 | 0.000 | 0.924 | 0.356 |
| Model 5 | 0.829 | 0.035 | 0.000 | 3.134 | 0.002 |
| Model 5+ CA | 0.825 | 0.036 | 0.000 | 3.261 | 0.001 |
| Model 5+ PhenoAge | 0.827 | 0.036 | 0.000 | 3.509 | 0.001 |

AUC: area under the curve; SE: standard error; PhenoAge: Phenotypic Age; CA: chronological age;

Model 4: a model that include lesion number, revascularization and disease count;

Model 5: a model that include all the variables in model 4, as well as gender, smoking, drinking, body mass index, serum uric acid, [creatine](javascript:;) [kinase](javascript:;), creatine phosphokinase isoenzyme, total cholesterol, triglycerides, low-density lipoprotein cholesterol, high-density lipoprotein cholesterol and N-terminal pro-brain natriuretic peptide.
